# Effect of Cr:Al Ratio on Corrosion Mechanism of Ni-Cr-Mo-Al Alloys in 3.5 wt.% NaCl Solution: Microstructure and Electrochemical and Passive Characteristics

**DOI:** 10.3390/ma18102177

**Published:** 2025-05-08

**Authors:** Chenggang Lian, Wei Xie, Huanjie Fang, Wenqian Wang, Jianhao Yu, Jicheng Li, Xiaodong He

**Affiliations:** 1 School of Marine Engineering Equipment, Zhejiang Ocean University, Zhoushan 316022, China; lianchenggang@nimte.ac.cn; 2 State Key Laboratory of Advanced Marine Materials, Ningbo Institute of Materials Technology and Engineering of the Chinese Academy of Sciences, No. 1219 Zhongguan West Road, Ningbo 315201, China; wangwenqian@nimte.ac.cn (W.W.); yujianhao@nimte.ac.cn (J.Y.); lijicheng@nimte.ac.cn (J.L.); hexiaodong@nimte.ac.cn (X.H.); 3 Qianwan Institute of CNITECH, Zhongchuang 1st Road, Zhongchuang Park, Qianwan New Area, Ningbo 315336, China

**Keywords:** marine environment, nickel-based alloy, Cr:Al ratio, electrochemical tests, passive behavior

## Abstract

In this study, the microstructure and electrochemical and passive characteristics of NiCr_25.2−x_MoAl_x_ (x = 0, 1.25, 2.5, and 5 mol.%) alloys were investigated. The results show that Ni-Cr-Mo-Al alloys with varying Cr:Al ratios both had a single FCC structure without any second structure precipitates, and decreases in dislocation density and grain size were observed as the Al content in NiCrMoAl alloys increased. It was found from the electrochemical results that the NiCr_23.95_MoAl_1.25_ alloys had the maximum radius for a semicircle and the lowest *I*_corr_, indicating an enhanced anti-corrosion performance (R_ct_: 8.08 ± 0.368 × 10^5^ Ω cm^2^, *I*_corr_: 1.05 ± 0.003 × 10^−7^ A/cm^2^). In this study, it was found that the anti-corrosion performance of the alloys had an approximate connection to the composition and density of passive films. Denser and more stable microstructures in NiCr_23.95_MoAl_1.25_ alloys were further proven by potentiostatic polarization tests and Mott–Schottky experiments, showing a lower stable current density and acceptor density (N_A_: 9.79 ± 0.4 × 10^−20^ cm^−3^). In addition, the results of XPS show that the Al_1.25_ specimen had the highest Cr_2_O_3_ in the passive film’s content among the NiCrMoAl alloys. Cr_2_O_3_ was the main component, suggesting an enhanced protective influence of passive film. The present study can offer guidance for the application of nickel-based alloys with high anti-corrosion resistance in marine environments.

## 1. Introduction

With the depletion of terrestrial resources caused by human over-exploitation, the development of marine resources is of particular importance, and it heavily relies on the rapid development of cutting-edge marine machinery. However, the marine environment is extremely harsh and complex. Seawater is widely considered to be a naturally strong electrolyte with high salt content, strong conductivity, and high microorganism content; therefore, a series of measures have been employed to ensure the successful long-term operation of marine equipment [1,2,3]. Among these numerous methods, nickel-based alloy is expected to become an effective solution because of its superior mechanical qualities and resistance to corrosion. In order to meet the requirements of working conditions, several types of nickel-based alloys, such as Monel [4], Inconel [5,6], Hastelloy [7], and Incoloy [8] have been developed and successfully applied in the industries of warships, gas turbines, and petrochemical engineering [9,10,11].

Nickel-based alloys have attracted widespread attention for their outstanding passivation ability [12,13,14,15,16]. When exposed to the marine environment, it is possible for the metal surface to quickly develop a protective oxide film [17,18], which can prevent the substrate from undergoing electrochemical reactions with the corrosive medium. The alloy’s composition affects the passivation film’s chemical makeup but is usually composed of oxides. The ability of nickel-based alloys to be corrosion-resistant can be effectively improved by adding a certain proportion of chromium (Cr), molybdenum (Mo), iron (Fe) and trace amounts of aluminum (Al), titanium (Ti), and other elements. For example, the passive film’s composition with more Cr_2_O_3_ can be promoted by introducing chromium (Cr), while the addition of molybdenum (Mo) into nickel-based alloys can improve pitting resistance and resistance to corrosion, particularly in acidic situations, which results from the makeup of oxides containing Mo^6+^ [19]. Consequently, the versatility of nickel-based alloys benefits from the incorporation of elements like Cr and Mo, enabling their application in complex corrosive conditions. However, when the amount of Mo is excessive, the σ phase is inevitably precipitated, causing decreased resistance to corrosion [20]. The present study focused on the density and stability of a passive film on the alloy surface, which has been widely recognized by researchers as a critical factor [21]. The dense passive film formed on the alloy surface, typically 1–3 nm thick, was identified as a key contributor to the alloy’s outstanding corrosion resistance.

Furthermore, many studies have explored the impact of Al addition on the mechanical qualities and resistance to corrosion of passive films. Pan et al. [22] examined how the addition of Al affects CoNiVAl_x_ medium-entropy alloy’s microstructure and resistance to corrosion, and Al addition was demonstrated to positively influence resistance to corrosion. This investigation further proposed that with Al addition, the passive film’s stability and protective qualities were improved when the oxide content increased and the concentration of defects decreased. The corrosion resistance ability of Al_x_CrFeNi_3-x_ high-entropy alloys with different Al elements was also investigated, employing electrochemical tests by Zhao et al. [23] Their findings were contrary to previous studies, and a decrease in the passive film’s stability was found as the Al content increased. Inconel 625 coatings with different Al contents were prepared by Feng et al. [24] Phase composition analysis showed that the addition of Al formed a body-centered cubic (BCC) phase, increasing the coatings’ hardness and wear resistance. In addition, an enhanced corrosion resistance was found with the addition of trace amounts of Al (wt.% 2.5%), but decreased as the Al content increased beyond this threshold. It is worth noting that when Al and Cr are added into nickel-based alloys, it is possible to see the development of an oxide layer, and the thermal stability of Al_2_O_3_ film was superior to that of Cr_2_O_3_ [25]. However, its corrosion resistance at room temperature has not been extensively studied.

To this end, this work systematically examined the influence of Cr:Al ratio on the corrosion mechanism of NiCr_25.2-x_MoAl_x_ (x = 0, 1.25, 2.5, 5 at%) alloys in 3.5 wt.% NaCl electrolyte solution. Specifically, electrochemical tests like the electrochemical impedance spectroscopy curve (EIS) and potentiodynamic polarization curve methods, were employed to evaluate electrochemical property of NiCrMoAl alloys with different Cr:Al ratio. In addition, constant potential polarization, Mott–Schottky (M-S) curve, and X-ray photoelectron spectroscopy (XPS) method were employed to study the characteristics of passive film. This study can offer valuable guidance for designing alloys with good corrosion resistance in marine environments.

## 2. Experimental Procedures

### 2.1. Sample Preparation

Mixtures of high-purity metals (purity > 99.95%) were arc-melted to create alloy ingots with different aluminum (Al) percentages; all the raw materials (Linyiyan Innovative Materials Technology Co., Ltd., Linyi, China) were irregular particles of about 3 mm in size. To avoid contamination from leftover oxygen and nitrogen, the entire procedure was conducted in an argon environment that absorbed titanium. To minimize compositional inhomogeneity caused by the volatilization of high-melting-point elements (Mo and Cr) and low-melting-point elements (Al), Ni particles were placed as protective layers on both the top and bottom of the material stack; these ingots were remelted at least five times while being stirred by a magnetic device, and then suction cast using a water-cooled copper mold into bulk samples measuring 60 × 20 × 6 mm^3^. After that, the castings were chopped into cuboid samples measuring 10 × 10 × 3 mm^3^. All the alloy samples were ground successively using SiC sandpaper (400#, 800#, 1200#, 2000#, and 3000#), polished using a polishing compound to a thickness of 1 µm to observe their microstructural characteristics, and then further polished using 0.05 µm silica suspension for electron backscatter diffraction (EBSD) analysis. Other copper wires were connected with the alloys with regard to the specimens prepared for electrochemical tests, and afterwards, the specimens were covered with epoxy resin, exposing just 1 cm^2^. The chemical compositions of NiCr_25.2−x_MoAl_x_ (x = 0, 1.25, 2.5, and 5 mol.%) alloys are listed in Table 1. For simplicity, the NiCrMoAl alloys with varying Cr:Al ratios are referred to as Al_0_, Al_1.25_, Al_2.5_, and Al_5_ in the following manuscript.

### 2.2. Electrochemical Measurements

All electrochemical tests, including dynamic potential polarization, constant potential current transient, and capacitance measurements, were conducted utilizing the Gamry workstation (American, Reference 620), and a regular three-electrode test setup, consisting of the counter electrode (in platinum, 10 × 10 × 0.2 mm^3^), an Ag/AgCl reference electrode (KCl saturated), 300 mL of 3.5 wt.% NaCl electrolyte solution, and the sample serving as the working electrode. All potentials were set relative to the Ag/AgCl reference electrode. Before testing, constant potential polarization was performed for 300 s at −0.5 V to reduce the effect of the oxide film.

To ensure that the system had stabilized, an open-circuit potential test was conducted for an hour before the EIS test, which was further confirmed by an allowable fluctuation over 10 min of no greater than ±5 mV. With an AC perturbation amplitude of 10 mV in a frequency range of 10^2^ kHz to 10 mHz, the EIS test was carried out. ZSimpWin (version 3.60) software was then applied to examine the EIS results. Subsequently, at a scan rate of 60 mV/min, a dynamic potential polarization test was conducted, beginning at −0.46 V until the polarization current density reached 2 mA/cm^2^.

A Mott–Schottky curve was applied to examine the passive film’s semiconductor characteristics, using a 50 mV/s scan rate, *E*_b_ − 0.2 V scan range, and a frequency of 1 kHz. Prior to the test, a constant potential polarization lasting for 600 s at −0.5 V was strictly necessary to remove any oxide film that may have formed. To create a stable oxide film in a 3.5 wt.% NaCl electrolyte solution, 18,000 s of polarization at 0.2 V was then carried out. All electrochemical tests were carried out at least 3 times to guarantee the accuracy and repeatability of the results.

### 2.3. Characterization

Utilizing X-ray diffraction (XRD), the alloy’s phase structure and the 2θ range of 20° to 100° were determined. To grasp the crystallographic data, including dislocation density and the size and orientation of the grains, a Gemini 300 scanning electron microscope system equipped a QUANTAX EBSD probe (Bruker, Billerica, MA, USA) was adopted. The resultant data were further analyzed using AZtecCrystal (version 3.1) software. Moreover, X-ray photoelectron spectroscopy (XPS, Axis Supra, Kratos, Japan) was used to describe the composition of the passive film that resulted from constant potential polarization. The XPS date was calibrated using the general peak with (C 1_s_, 284.8 eV), and the findings were analyzed using Avantage (version 5.9922) software, with the Shirley model applied for background subtraction.

## 3. Results and Discussion

### 3.1. Microstructure Analysis

**Figure 1:**
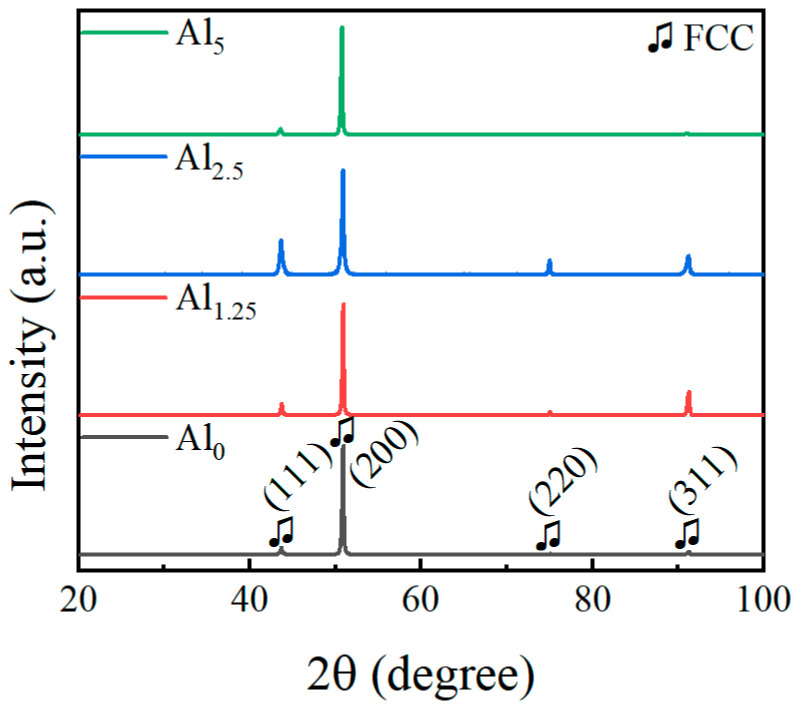
XRD patterns of the Al_0_, Al_1.25_, Al_2.5_, and Al_5_ alloys.

The XRD spectrum of the cast NiCr_25.2−x_MoAl_x_ alloy is shown in Figure 1. All characteristic peaks were corresponded to the single FCC structure, with peaks at (111), (200), (220), and (311), indicating that NiCr_25.2−x_MoAl_x_ was composed of a single FCC phase. This result was consistent with typical NiCrMo alloys [26].

**Figure 2:**
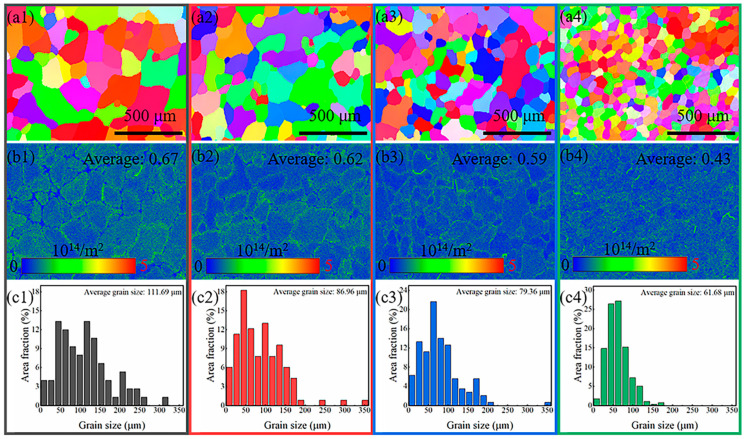
EBSD results including (**a1**–**a4**) IPF maps, (**b1**–**b4**) GND and (**c1**–**c4**) grain size of the (**a1**–**c1**) Al_0_, (**a2**–**c2**) Al_1.25_, (**a3**–**c3**), Al_2.5_, and (**a4**–**c4**) Al_5_ alloys.

The EBSD data presented in Figure 2 show the inverse pole figure (IPF), dislocation density (GND), and the average grain size for Al_0_, Al_1.25_, Al_2.5_, and Al_5_ alloys. From the IPF maps in Figure 2(a1–a4), it can be observed that the alloy grains with varying Cr:Al ratio exhibited uniform equiaxed structures. The GND maps in Figure 2(b1–b4) further revealed that the dislocation density decreased with the continuous addition of Al content. This phenomenon can be attributed to the large atomic radius and low elastic modulus of Al, which caused a local stress field that inhibited dislocation movement and generation, thus leading to a reduction in dislocation density [23]. Some studies [7,27] proposed that an increased dislocation density would enhance the passive film’s nucleation sites, thereby promoting its formation. In addition, Figure 2(c1–c4) gives the G-S statistics of the NiCrMoAl alloys, and the results demonstrated that the average grain size reduced as the amount of Al rose, which was consistent with the reports of other studies [28]. However, it is worth pointing out that grain size is negatively correlated with corrosion resistance [21,29]. That is, dislocation density and grain size have the opposing effects on corrosion resistance, and corrosion resistance falls as grain size decreases. Therefore, it is difficult to accurately speculate the anti-corrosion resistance of NiCrMoAl alloys with varying Cr:Al ratio based solely on their grain microstructure.

### 3.2. Electrochemical Characteristic Analysis

**Figure 3:**
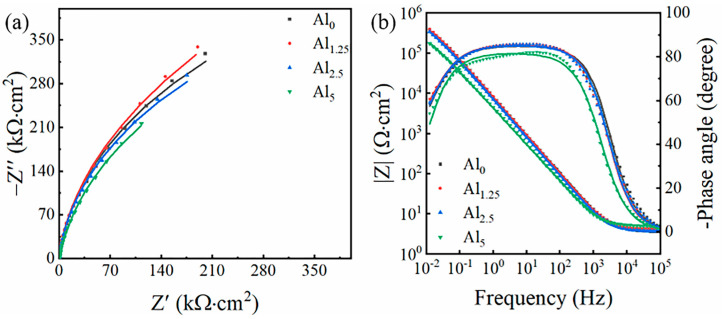
(**a**) Nyquist plot and (**b**) Bode plot of the Al_0_, Al_1.25_, Al_2.5_, and Al_5_ in 3.5 wt.% NaCl electrolyte solution. The fitting curves are shown by solid lines.

**Figure 4:**
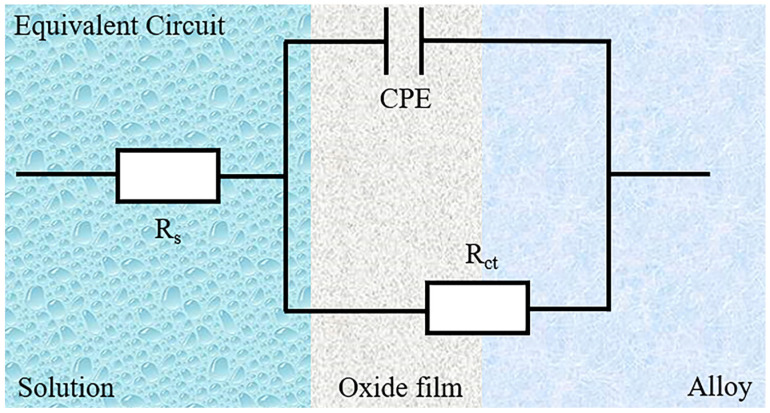
The equivalent circuit applied in the EIS date fitting.

EIS was employed for comprehensive evaluation in order to assess the corrosion resistance of the alloy samples with varying Cr:Al ratios. As shown in Figure 3a, the Nyquist plot exhibited a classic capacitive semicircle, which can be attributed to the formation of protective oxide film on the sample surface after rapid oxidation due to pre-treatment of open-circuit potential. The Nyquist plot displayed a classic capacitive semicircle, as seen in Figure 3a. This is because of the open-circuit voltage test, causing a protective oxide film to form on the sample surface following rapid oxidation. Additionally, the radius of the semicircle followed the order Al_1.25_ > Al_0_ > Al_2.5_ > Al_5_, indicating an enhanced resistance to electrochemical corrosion when the content of Al reached to 1.25 mol.%. In the Bode plot shown in Figure 3b, a broad peak can be seen in the range of 10^0^–10^3^ Hz, suggesting the oxide film’s capacitive qualities on the sample surface. Figure 4 displays the circuit model that was utilized to fit the data, with *R*_s_ standing for the electrolyte solution’s resistance, and CPE is used to denote the constant phase element that reflecting the capacitance of the oxide film on the alloy’s surface [28,29]. This CPE is connected in parallel with *R*_ct_, the charge transfer resistance, which indicates the resistance encountered by corrosive ions as they pass through the oxide film to reach the alloy interface. The impedance value of CPE can be calculated using the following formula:

(1) $$Z_{CPE}=\frac{1}{Q{\left(j\omega \right)}^{n}}$$

where *Z_CPE_* is the impedance value, Q is the admittance value, ω is the angular frequency, j is an imaginary number, and *n* (0 ≤ *n* ≤ 1) is the fitting parameter. *n* = 0 represents pure resistance, n = 0.5 represents Warburg impedance, and *n* = 1 represents an ideal capacitor. The parameters obtained from fitting the test results using ZSimpWin software are listed in Table 2, and a small value of chi-square (χ^2^) (on the order of 10^−4^) indicates a good fit. In Table 2, the CPE_dl_ value of the Al_1.25_ sample was the lowest, while that of the Al_5_ sample was the highest, indicating that the Al_1.25_ sample exhibited the best corrosion resistance, whereas the Al_5_ sample showed the poorest corrosion resistance. Moreover, the R_ct_ value of the Al_1.25_ sample was the highest, suggesting that its oxide film provided the most effective protection. These results demonstrate that the addition of a small amount of Al significantly enhanced the corrosion resistance of the alloy.

**Figure 5:**
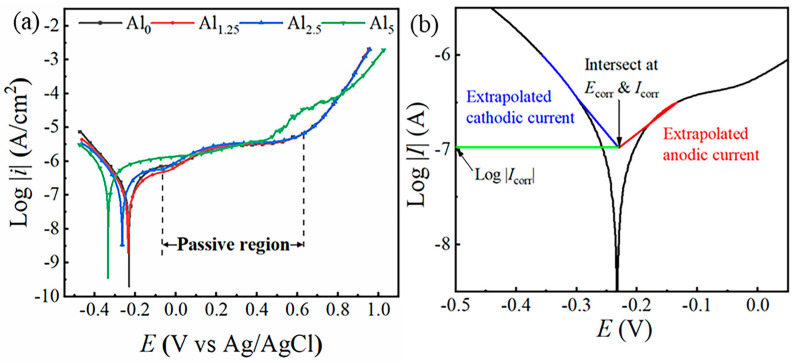
(**a**) Dynamic potential polarization curves of Al_0_, Al_1.25_, Al_2.5_, and Al_5_ specimens in 3.5 wt.% NaCl electrolyte solution and (**b**) Tafel extrapolation.

Figure 5a plots the dynamic potential polarization of NiCr_25.2−x_MoAl_x_ alloys. The polarization curves revealed that all samples exhibited similar characteristics, including visible passivation regions [30]. As the voltage increased in the early dissolution zone, the corrosion current density (*I*_corr_) increased quickly, but this behavior was mitigated in the subsequent transition zone. Because of the passive film’s protective impact, the growth rate of the corrosion current was lowered in the passivation zone, though it worsened in the over-passivation region. The Tafel extrapolation method was used to fit the pertinent values from the polarization curves (Figure 5b) [31], and other values are compiled in Table 3, including the pitting potential (*E*_p_), passive current density (*I*_p_), corrosion current density (*I*_corr_), and self-corrosion potential (*E*_corr_) for each alloy. Among all samples, the highest *E*_corr_ was found in the Al_0_ specimen, and as Al content increased, *E*_corr_ gradually decreased, which was likely due to the lower electronegativity of Al. However, it was notable that the Al_1.25_ specimen unexpectedly showed the lowest *I*_corr_, which was in agreement with the results of EIS. Therefore, it can be concluded that the trace amounts of introduced Al can effectively improve the anti-corrosion performance of NiCr_25.2−x_MoAl_x_ alloys, but further increases in Al content resulted in a weak corrosion resistance.

### 3.3. Passivation Characteristic Analysis

**Figure 6:**
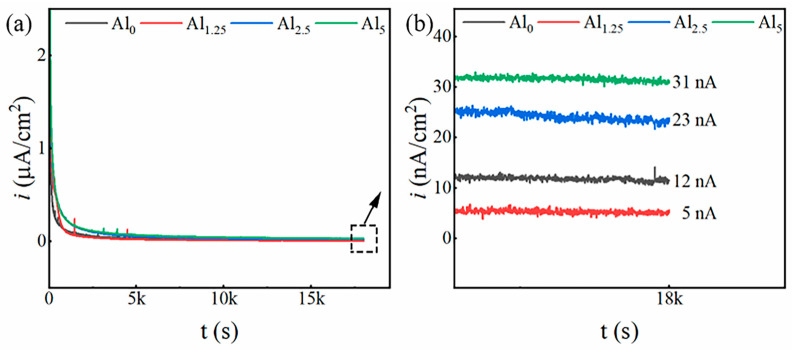
(**a**) Current–time curves and (**b**) the results for 18,000 s at an imposed potential of Al_0_, Al_1.25_, Al_2.5_, and Al_5_ alloys in the 3.5 wt.% NaCl electrolyte solution.

To explore the possible causes for different corrosion resistance, passivation behavior of the samples with different Cr:Al ratios was invested. It is well acknowledged that the density of the passive film plays a significant role in determining how resistant an alloy is to corrosion. A stable passivation potential was applied for 5 h of constant potential polarization, with current density measured over time, and the results of potentiostatic polarization are shown in Figure 6, revealing a sharp decrease in current density during the test’s first phase, credited to the passive film’s quick formation [32]. Throughout the polarization process, sudden fluctuations in current density were also observed, which were likely caused by the localized dissolution of the passive film [33]. After 5 h of constant potential polarization, the current density reached a stable state. The steady-state current densities were found to follow the order of 5 nA (Al_1.25_) < 12 nA (Al_0_) < 23 nA (Al_2.5_) < 31 nA (Al_5_). Notably, the Al_1.25_ sample exhibited much smaller dispersion and a lower passivation current density, demonstrating the formation of a denser and more stable passive film.

The connection between the imposed voltage (*E*) and the space charge capacitance (*C*) was described by the M-S equation, as presented below [34,35].

(2) $$\frac{1}{C^{2}}=\pm \frac{2}{\varepsilon \varepsilon_{0}eN}\left(E-E_{FB}-\frac{KT}{e}\right)$$

where ***ε*** represented the vacuum permittivity (8.854 × 10^−14^ F/cm), while ***ε***_0_ denoted the relative permittivity (typically 12). *E* is the applied external potential, and *E*_FB_ referred to the flat band potential (V). In addition, the electron charge was given as e = 1.602 × 10^−19^ C, and *N* corresponded to the carrier defect concentration (cm^−3^). *K* and *T* denoted the Boltzmann constant and temperature (K). Generally, the defect concentration can be inferred from the slope of the M-S curve. A higher defect concentration would result in a smaller absolute value of slope, showing a slower variation in the curve. Furthermore, the slope’s indication could be used to identify the type of defect. The passive film displayed p-type semiconductor characteristics when the slope was negative, where the carrier defects corresponded to cation vacancies, denoted as *N*_A_ [36].

**Figure 7:**
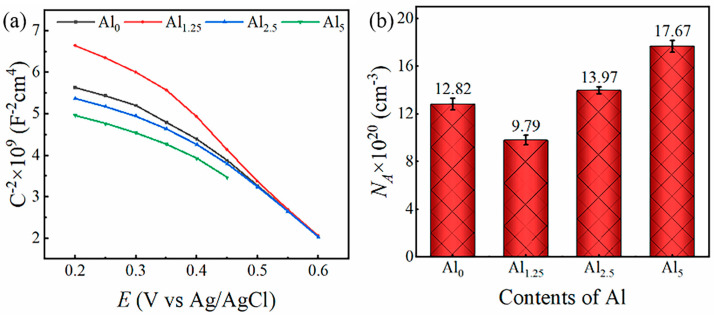
(**a**) M-S curves of Al_0_, Al_1.25_, Al_2.5_, and Al_5_ in 3.5 wt.% NaCl electrolyte solution and (**b**) the calculated values of the NA according to the M-S curves.

Figure 7a shows the Mott–Schottky curves for NiCr_25.2−x_MoAl_x_ alloys, illustrating their passive films’ semiconductor characteristics. Every curve exhibited a negative slope, confirming the passive film’s p-type semiconductor properties, with cation vacancies as the predominant point defects [37,38]. Significant variations in the curves’ slope were noted as a result of the passive film’s non-uniform acceptor density distribution [39]. The acceptor density (*N*_A_) of each sample was determined by calculating the linear region’s slope, as shown in Figure 7b. The numerical range of *N*_A_ was found to be between 10^20^ and 10^21^, which aligned with the defect concentration of conventional stainless steels [40,41]. The conclusion that the Al_1.25_ sample had a lower defect density was demonstrated again by Mott–Schottky experiments. With the further addition of Al, *N*_A_ significantly increased, and accordingly, the ability to inhibit the passage of electrolytes weakened.

**Figure 8:**
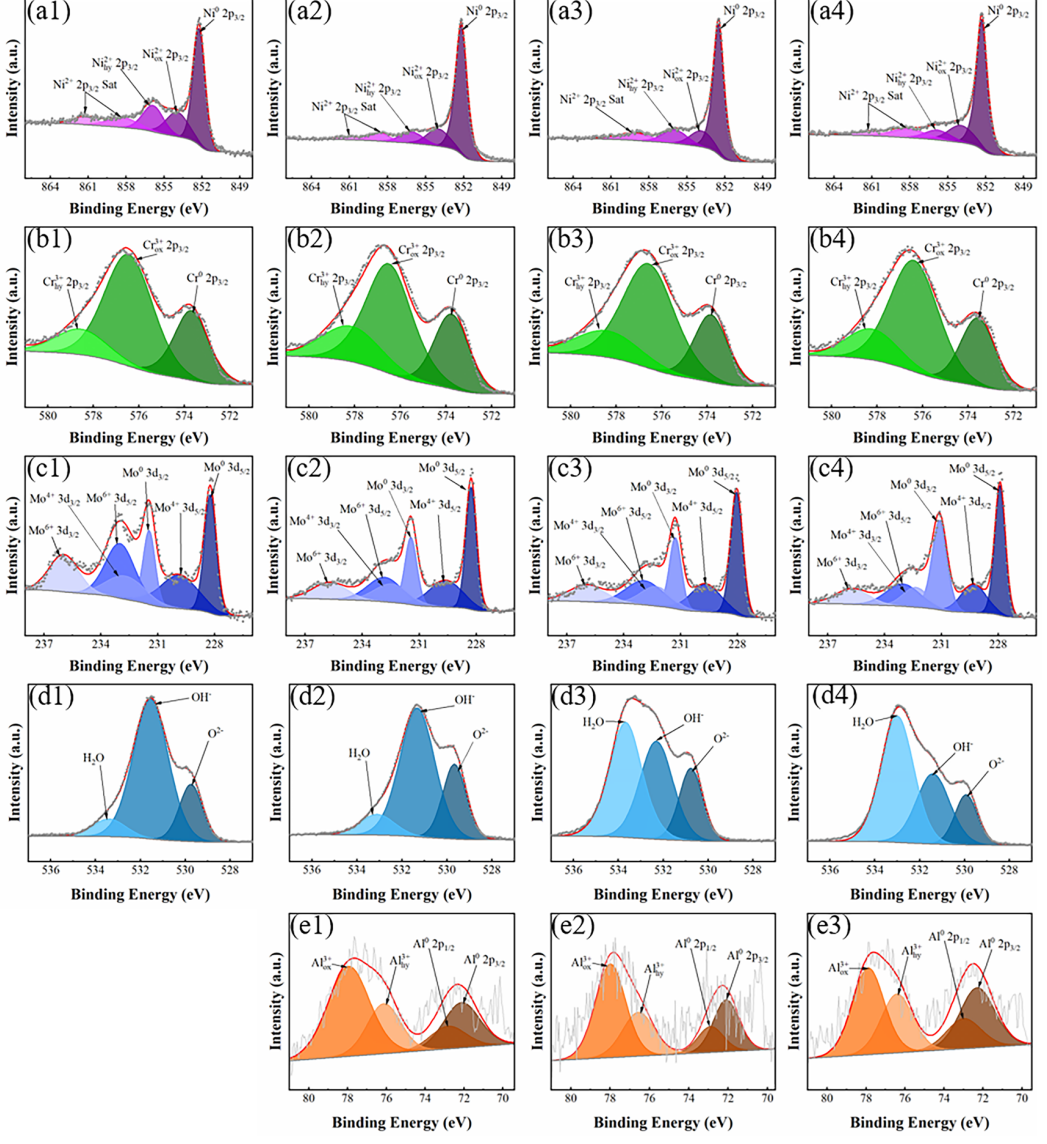
XPS spectrums of (**a1**–**a4**) Ni 2p, (**b1**–**b4**) Cr 2p, (**c1**–**c4**) Mo 3d, (**d1**–**d4**) O 1s, and (**e1**–**e3**) Al 2p for the passive film formed on (**a1**–**d1**) Al_0_, (**a2**–**d2**) Al_1.25_, (**a3**–**d3**), Al_2.5_, and (**a4**–**d4**) Al_5_ in 3.5 wt.% NaCl electrolyte solution.

Additionally, the composition of passive films directly influenced its protective performance [42]. Figure 8 presents the comprehensive spectra of Ni 2p, Cr 2p, Mo 3d, and O 1s obtained through fitting analysis. The Ni 2p spectrum was discovered to have four component peaks, corresponding to Ni metal, Ni_ox_^2+^, Ni_hy_^2+^, and Ni^2+^ Sat, respectively. Among these, the peak associated with metallic nickel was the most prominent. The main cause of this was that, during the characterization procedure, some nickel oxide was reduced to metallic Ni. Additionally, the higher electronegativity of metallic nickel compared to Cr and Al resulted in most of the oxygen reacting with Cr and Al during the oxidation process, leaving the passive film with a greater concentration of metallic nickel [43,44]. Furthermore, the Cr 2p spectrum could be decomposed into the peaks that represent metal Cr^0^, Cr_ox_^3+^, and Cr_hy_^3+^. The results indicate that variations in the types of chromium shown in the passive film were not substantially impacted by the Cr:Al ratio. In Figure 8(c1–c4), Mo^0^ 3d_5/2_, Mo^0^ 3d_3/2_, Mo^4+^ 3d_5/2_, Mo^4+^ 3d_3/2_, Mo^6+^ 3d_5/2_, and Mo^6+^ 3d_3/2_ are the peaks that were identified in the Mo 3d spectrum. Notably, metallic Mo was the dominant species, suggesting that Mo was less likely to undergo passivation in a neutral solution, which resulted in a relatively low oxide content. Figure 8(d1–d4) presents the O 1s spectrum, which can be categorized into three characteristic peaks for H_2_O, OH^−^, and O^2−^, corresponding to the passive film’s bound water, metal hydroxides, and oxides, respectively. Bound water is a critical component of the passive film, as it can interact with the solution’s metal ions, facilitating the formation of additional oxide layers [45,46]. In addition, the Al 2p spectrum, as shown in Figure 8(e1–e3), can be decomposed into peaks for Al^0^ 2p_3/2_, Al^0^ 2p_1/2_, Al_ox_^3+^ and Al_hy_^3+^. Some studies proposed that alumina (Al_2_O_3_) can inhibit alloy oxidation under high-temperature conditions; however, the porous nature of alumina raised concerns about its effectiveness in strengthening the alloys’ resistance to corrosion in certain environments [47,48]. The detailed parameters are provided in the Supplementary Material.

**Figure 9:**
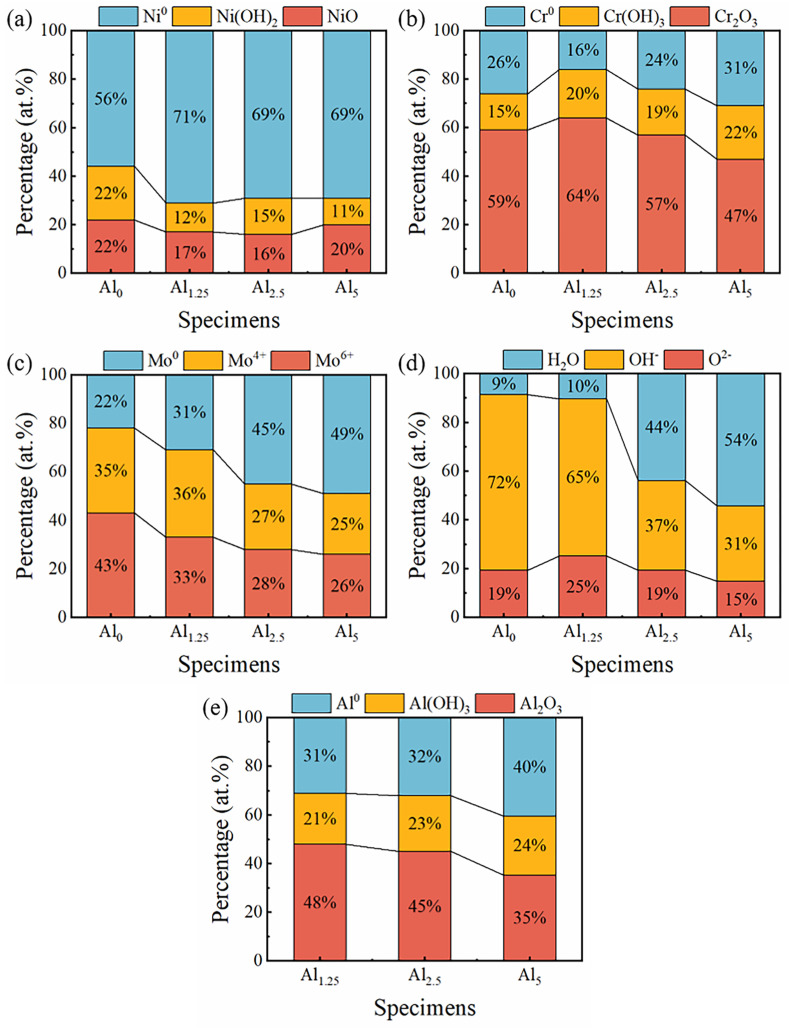
The atomic percentages of the component peaks to the total intensity of (**a**) Ni 2p, (**b**) Cr 2p, (**c**) Mo 3d, (**d**) O 1s, and (**e**) Al 2p in the passive film of Al_0_, Al_1.25_, Al_2.5_, and Al_5_ alloys in 3.5 wt.% NaCl electrolyte solution.

By calculating the area of each fitting peak, the percentage of each valence state for each element can be intuitively quantified and compared. For NiCr-based alloys, the presence of Cr_2_O_3_ is principally responsible for the passive film’s protective action. Among these NiCrMoAl alloys, the Al_1.25_ sample has the highest Cr_2_O_3_ concentration, as seen in Figure 9b. In addition, the O^2−^ contents in NiCr_25.2−x_MoAl_x_ samples were 19%, 25%, 19%, and 15%, respectively. When measuring the oxygen content, the oxide content of the passivation film was relatively reliable. The highest content of O^2−^ in the Al_1.25_ sample was significantly higher, suggesting that the addition of trace amounts of Al encourage the film’s oxide development. A previous study [27] also found that increasing the Al content can improve the corrosion resistance of FeCoCrNiAl_x_ by simultaneously promoting the formation of Cr_2_O_3_ in the passive film, making it thicker in the H_2_SO_4_ solution.

## 4. Conclusions

This study systematically investigated the effect of Cr:Al ratio on the corrosion mechanism of NiCr_25.2-x_MoAl_x_ (x = 0, 1.25, 2.5 and 5 mol.%) alloys in 3.5 wt.% NaCl solution. Specifically, electrochemical tests were employed to evaluate electrochemical behavior of NiCrMoAl alloys with different Cr:Al ratios. In addition, constant potential polarization, M-S curve, and XPS were used to study the characteristics of passivation film. The following conclusions were drawn:

(1) The Ni-Cr-Mo-Al alloys with varying Cr:Al ratio both exhibited a single face-centered cubic (FCC) phase without any second-phase precipitates. In addition, uniform equiaxed structures were observed in all specimens; however, the results of EBSD showed a decreased dislocation density and grain size as Al content in NiCrMoAl alloys increased. The decrease in grain size was beneficial to the corrosion resistance of the alloy, while a decreasing dislocation density affected the nucleation and growth of passivation film, thereby decreasing anti-corrosion performance.
(2) The corrosion resistance of the alloy samples with different Cr:Al ratio was compared by electrochemical methods, including EIS and PDP. A maximum radius of a semicircle was found in the Al_1.25_ specimen on EIS test, whereas the highest ***E***_corr_ was found in the Al_0_ specimen, and as Al content increased, ***E***_corr_ gradually decreased. However, the Al_1.25_ specimen unexpectedly showed the lowest ***I***_corr_. The results of EIS and PDP both indicated that the Al_1.25_ sample exhibited the best electrochemical performance (R_ct_: 8.08 ± 0.368 × 10^5^ Ω cm^2^, I_corr_: 1.05 ± 0.003 × 10^−7^ A/cm^2^).
(3) The density of passivation film has an essential influence on the corrosion resistance of alloys. In the potentiostatic polarization test, steady-state current densities were observed to follow the order 5 nA (Al_1.25_) <12 nA (Al_0_) <23 nA (Al_2.5_) < 31 nA (Al_5_), indicating that the passive film with a denser and more stable microstructure was formed in Al_1.25_ specimen. The similar conclusion was demonstrated again in Mott–Schottky experiments. When the Al content was 1.25 mol.%, the amount of ***N***_A_ was 9.79 × 10^20^ cm^−3^, which was lowest among these alloys.
(4) The composition of a passive film was another important factor affecting its protective performance. It can be found from XPS results that the content of Cr_2_O_3_ in the passive film, the main component of the passive film, first increased and then decreased. Among these Ni-Cr-Mo-Al alloys, the Al_1.25_ specimen had a highest Cr_2_O_3_ content, implying an enhanced protective effect of the passive film.

## Figures and Tables

**Table 1:** Chemical compositions of NiCrMoAl alloys with varying Cr:Al ratio.

Alloy		Elements			
		Ni	Cr	Mo	Al
Al_0_	Nominal (at.%)	71.7	25.2	3.1	-
	Weight (wt.%)	72.4	22.5	5.1	-
Al_1.25_	Nominal (at.%)	71.7	23.95	3.1	1.25
	Weight (wt.%)	72.8	21.5	5.1	0.6
Al_2.5_	Nominal (at.%)	71.7	22.7	3.1	2.5
	Weight (wt.%)	73.1	20.5	5.2	1.2
Al_5_	Nominal (at.%)	71.7	20.2	3.1	5
	Weight (wt.%)	73.9	18.5	5.2	2.4

**Table 2:** Equivalent circuit model fitting parameters based on the EIS results.

Alloy	*R*_s_ (Ω·cm^2^)	CPE × 10^−5^ (Ω^−1^ cm^−2^ s^n^)	*n*	*R*_ct_ × 10^−5^ (Ω·cm^2^)
Al_0_	6.788 ± 0.04	2.23 ± 0.02	0.9417 ± 0.002	7.66 ± 0.362
Al_1.25_	6.276 ± 0.04	2.22 ± 0.01	0.9461 ± 0.001	8.08 ± 0.368
Al_2.5_	6.807 ± 0.03	2.53 ± 0.02	0.9381 ± 0.002	6.95 ± 0.356
Al_5_	6.614 ± 0.05	3.52 ± 0.03	0.8961 ± 0.002	6.26 ± 0.522

**Table 3:** Electrochemical values fitting from the test results of Al_0_, Al_1.25_, Al_2.5_, and Al_5_ alloys in 3.5 wt.% NaCl electrolyte solution.

Alloy	*I*_corr_ × 10^−7^ (A)	*E*_corr_ (V)	*I*_p_ × 10^−6^ (A)	*E*_b_ (V)
Al_0_	1.55 ± 0.004	−0.23 ± 0.005	3.14 ± 0.002	0.61 ± 0.02
Al_1.25_	1.05 ± 0.003	−0.24 ± 0.003	3.01 ± 0.002	0.61 ± 0.01
Al_2.5_	1.58 ± 0.005	−0.26 ± 0.004	3.50 ± 0.004	0.61 ± 0.01
Al_5_	2.21 ± 0.002	−0.33 ± 0.002	6.17 ± 0.003	0.46 ± 0.03

## Data Availability

The data presented in this study are available on request from the corresponding author due to privacy.

## Supplementary Materials

The following supporting information can be downloaded at: https://www.mdpi.com/article/10.3390/ma18102177/s1. Table S1: XPS fitting parameters of components on the Ni 2p, Cr 2p, Mo 3d, O 1s, and Al 2p spectra.
