# Supplementary material for: Effect of Cr:Al Ratio on Corrosion Mechanism of Ni-Cr-Mo-Al Alloys in 3.5 wt.% NaCl Solution: Microstructure and Electrochemical and Passive Characteristics
Source: Materials (Basel). 2025 May 8;18(10):2177. doi: 10.3390/ma18102177 (PMC12113175; doi:10.3390/ma18102177)
Supplement: Supplementary file 1 [file materials-18-02177-s001.zip › materials-3595972-supplementary.pdf]

SUPPORTING INFORMATION

Effect of Cr:Al Ratio on Corrosion Mechanism of Ni-Cr-Mo-Al Alloys in 3.5 wt.% NaCl Solution: Microstructure and Electrochemical and Passive Characteristics

Chenggang Lian [1,2,3], Wei Xie [1,*], Huanjie Fang [2,3,*], Wenqian Wang [2,3], Jianhao Yu [2,3], Jicheng Li [2,3], Xiaodong He [2,3]

[1] School of Marine Engineering Equipment, Zhejiang Ocean University, Zhoushan 316022, China; lianchenggang@nimte.ac.cn

[2] State Key Laboratory of Advanced Marine Materials, Ningbo Institute of Materials Technology and Engineering, Chinese Academy of Sciences, Ningbo 315201, China; wangwenqian@nimte.ac.cn (W.W.); yujianhao@nimte.ac.cn (J.Y.); lijicheng@nimte.ac.cn (J.L.); hexiaodong@nimte.ac.cn (X.H.)

[3] Qianwan Institute of CNITECH, Zhongchuang 1st Road, Zhongchuang Park, Qianwan New Area, Ningbo 315336, China

* Correspondence: weixie@zjou.edu.cn (W.X.); fanghuanjie@nimte.ac.cn (H.F.)

Table S1. XPS fitting parameters of components on the Ni 2p, Cr 2p, Mo 3d, O 1s, and Al 2p spectra.

Alloy	Peak	Assignment	E_b (eV)	FWHM (eV)
Al_0	Ni $2p_{3/2}$	Ni^0	852.23	1.12
		NiO	853.96	2.12
		$Ni(OH)_2$	855.87	1.92
	Cr $2p_{3/2}$	Cr^0	573.68	1.8
		Cr_2O_3	576.44	2.5
		$Cr(OH)_3$	578.55	2.68
	Mo 3d	Mo^0 $3d_{5/2}$	228.23	0.75
		Mo^0 $3d_{3/2}$	231.46	0.75
		Mo^{4+} $3d_{5/2}$	229.88	2.4
		Mo^{4+} $3d_{3/2}$	232.69	2.4
		Mo^{6+} $3d_{5/2}$	232.99	1.83
		Mo^{6+} $3d_{3/2}$	235.96	1.83

	O 1s	H_2O	533.4	1.73
		O^{2-}	529.74	1.22
		OH^-	531.54	1.85
	Ni $2p_{3/2}$	Ni^0	852.19	1.08
		NiO	854	2.12
		$Ni(OH)_2$	856.08	2.16
	Cr $2p_{3/2}$	Cr^0	573.62	1.86
		Cr_2O_3	576.48	2.58
		$Cr(OH)_3$	578.19	2.88
$Al_{1.25}$	Mo 3d	Mo^0 $3d_{5/2}$	228.28	0.65
		Mo^0 $3d_{3/2}$	231.45	0.75
		Mo^{4+} $3d_{5/2}$	229.6	2.12
		Mo^{4+} $3d_{3/2}$	232.82	2.12
		Mo^{6+} $3d_{5/2}$	232.82	2.12
		Mo^{6+} $3d_{3/2}$	235.71	2.12
$Al_{2.5}$	O 1s	H_2O	533.1	1.8
		O^{2-}	529.66	1.23
		OH^-	531.32	1.83
	Al 2p	Al^0 $2p_{1/2}$	72.82	2.2
		Al^0 $2p_{3/2}$	72.12	2.2
		Al_2O_3	77.94	2.54
		$Al(OH)_3$	76.13	2.03
	Ni $2p_{3/2}$	Ni^0	852.5	0.99
		NiO	853.84	2.12
		$Ni(OH)_2$	855.96	2.31
	Cr $2p_{3/2}$	Cr^0	573.83	1.76
		Cr_2O_3	576.57	2.68
		$Cr(OH)_3$	578.38	3.36
	Mo 3d	Mo^0 $3d_{5/2}$	228.04	0.75
		Mo^0 $3d_{3/2}$	231.28	0.75
		Mo^{4+} $3d_{5/2}$	229.68	1.92
		Mo^{4+} $3d_{3/2}$	232.28	1.92
		Mo^{6+} $3d_{5/2}$	232.91	2.31
		Mo^{6+} $3d_{3/2}$	235.96	2.31

	O 1s	H_2O	533.68	1.72
		O^{2-}	530.76	1.23
		OH^-	532.3	1.73
	Al 2p	Al^0 $2p_{1/2}$	72.89	1.54
		Al^0 $2p_{3/2}$	72.1	1.54
		Al_2O_3	77.98	1.81
		$Al(OH)_3$	76.56	1.92
Al_5	Ni $2p_{3/2}$	Ni^0	852.32	1.02
		NiO	853.96	2.4
		$Ni(OH)_2$	855.8	2.42
	Cr $2p_{3/2}$	Cr^0	573.56	1.79
		Cr_2O_3	576.36	2.5
		$Cr(OH)_3$	578.22	2.69
	Mo 3d	Mo^0 $3d_{5/2}$	227.91	0.66
		Mo^0 $3d_{3/2}$	231.11	0.93
		Mo^{4+} $3d_{5/2}$	229.3	1.58
		Mo^{4+} $3d_{3/2}$	232.35	1.58
		Mo^{6+} $3d_{5/2}$	233.03	2.12
		Mo^{6+} $3d_{3/2}$	235.73	2.12
	O 1s	H_2O	533	1.73
		O^{2-}	529.93	1.2
		OH^-	531.41	1.77
	Al 2p	Al^0 $2p_{1/2}$	72.99	2.12
		Al^0 $2p_{3/2}$	72.3	2.12
		Al_2O_3	77.89	1.92
		$Al(OH)_3$	76.41	1.92
